# Supplementary material for: Correlation of host inflammatory cytokines and immune-related metabolites, but not viral NS1 protein, with disease severity of dengue virus infection
Source: PLoS One. 2020 Aug 7;15(8):e0237141. doi: 10.1371/journal.pone.0237141 (PMC7413495; doi:10.1371/journal.pone.0237141)
Supplement: S1 Table — (DOCX) [file pone.0237141.s001.docx]

**S1 Table: Metabolites present in the serum and its regulation comparing DWOWS, DWWS and SD with HC.**

| Compound | Log FC ([DWOWS] vs [HC]) | Regulation ([DWOWS] vs [HC]) | Log FC ([DWWS] vs [HC]) | Regulation ([DWWS] vs [HC]) | Log FC ([SD] vs [HC]) | Regulation ([SD] vs [HC]) |
| --- | --- | --- | --- | --- | --- | --- |
| SM(d18:1/16:0) | 10.08594 | up | 1.2384071 | up | 6.026299 | up |
| SM(d16:1/18:1) | 6.0016937 | up | 6.8974295 | up | 6.496338 | up |
| 1-Linoleoylglycerophosphocholine | 7.42229 | up | 7.2415133 | up | 6.995461 | up |
| SM(d18:1/14:0) | 6.088464 | up | 6.9402256 | up | 6.435886 | up |
| PS(O-16:0/20:2(11Z,14Z)) | 5.962893 | up | 4.639504 | up | 4.016306 | up |
| PC(18:4(6Z,9Z,12Z,15Z)/18:1(11Z)) | 6.3992023 | up | 2.6399872 | up | 4.838449 | up |
| PC(13:0/0:0) | 6.8084083 | up | 6.8644648 | up | 6.699334 | up |
| PC(20:4(5Z,8Z,11Z,14Z)/0:0) | 7.251088 | up | 7.237234 | up | 7.107138 | up |
| PC(22:6(4E,7E,10E,13E,16E,19E)/0:0)[U] | 6.988765 | up | 7.157304 | up | 6.661316 | up |
| PS(O-18:0/20:3(8Z,11Z,14Z)) | 8.279184 | up | 6.3037643 | up | 4.008945 | up |
| PC(O-16:1(11Z)/2:0) Esi+15.371249 | 7.1879187 | up | 6.6485915 | up | 6.85951 | up |
| C27 H36 N14 O5 | 6.1224103 | up | 6.281932 | up | 6.471661 | up |
| 1-Linoleoylglycerophosphocholine Esi+13.36075 | 7.3936 | up | 7.232909 | up | 6.862653 | up |
| Estra-1,3,5(10)-triene-3,6alpha,17beta-triol triacetate | 6.86384 | up | 7.171279 | up | 5.603232 | up |
| PS(O-18:0/22:6(4Z,7Z,10Z,13Z,16Z,19Z)) Esi+30.26385 | 6.8113337 | up | 6.1035633 | up | 4.326638 | up |
| C8 H4 O3 | -3.4595242 | down | 6.769369 | up | 6.90314 | up |
| C19 H41 N O2 | 6.6074123 | up | 6.591388 | up | -4.63587 | down |
| Giganin | 3.8697987 | up | 3.706482 | up | 6.291902 | up |
| Stearamide | 1.5947561 | up | 6.50901 | up | 6.33808 | up |
| PS(O-18:0/20:3(8Z,11Z,14Z)) Esi+31.897125 | 6.2908278 | up | 5.217903 | up | 2.516715 | up |
| PI-Cer(t18:0/18:0) | 4.7934847 | up | 4.751927 | up | 9.86359 | up |
| Palmitic amide | 4.4670935 | up | 6.8822713 | up | 6.647247 | up |
| PC(P-18:0/20:4(5Z,8Z,11Z,14Z)) | 3.484333 | up | 7.533722 | up | 11.95465 | up |
| Chamuvaritin | 7.8328753 | up | 7.4007277 | up | 5.682369 | up |
| Labda-7,14-dien-6-one, 13-hydroxy | 2.2996478 | up | 7.3069954 | up | 7.446309 | up |
| ε-Caprolactam | 8.26923 | up | 7.820588 | up | 8.092072 | up |
| C32 H58 O6 | 7.144595 | up | 6.6769123 | up | 6.819612 | up |
| C28 H52 O5 | 6.3214107 | up | 6.566876 | up | 6.706885 | up |
| N-docosahexaenoyl GABA | 8.088014 | up | 7.837258 | up | 7.7044 | up |
| PC(16:0/20:4(5Z,8Z,10E,14Z)(12OH[S])) Esi+32.41271 | 5.1533327 | up | 4.657929 | up | -9.36767 | down |
| alpha-Methylstyrene | 7.018153 | up | 7.3240037 | up | 5.683378 | up |
| C24 H33 N10 O7 | 6.5256124 | up | 6.18477 | up | 6.339412 | up |
| PS(O-20:0/22:6(4Z,7Z,10Z,13Z,16Z,19Z)) | 4.8150177 | up | 6.323385 | up | -0.00316 | down |
| SM(d16:1/17:0) | -1.4326453 | down | 6.9545584 | up | 6.468743 | up |
| SM(d18:0/16:0) | 7.0818596 | up | 12.240127 | up | 8.781132 | up |
| PE(17:2(9Z,12Z)/22:4(7Z,10Z,13Z,16Z)) | 4.2887387 | up | 7.7015934 | up | 3.609073 | up |
| PC(18:1(4Z)/18:1(4Z)) | 4.5157375 | up | 5.617967 | up | 9.184445 | up |
| C22 H35 N14 O4 | 7.7071333 | up | 6.4750576 | up | 6.750144 | up |
| C22 H45 O14 | 7.3345385 | up | 6.6823435 | up | 6.431593 | up |
| C36 H64 N3 O5 | 7.7768364 | up | 6.751317 | up | 6.7833 | up |
| PC(13:0/0:0) Esi+14.172747 | 6.921403 | up | 6.948127 | up | 6.720923 | up |
| N-dodecanoyl-L-Homoserine lactone-3-hydrazone-biotin | 6.903036 | up | 6.793583 | up | 6.675522 | up |
| C30 H59 N4 O9 | 9.292707 | up | 8.063892 | up | 10.05362 | up |
| PS(O-20:0/22:6(4Z,7Z,10Z,13Z,16Z,19Z)) Esi+33.30551 | 6.539532 | up | 5.468504 | up | 3.391531 | up |
| C30 H57 N O9 | 7.651404 | up | 5.0975285 | up | 2.404843 | up |
| PC(18:3(6Z,9Z,12Z)/18:0) Esi+33.603256 | 8.933014 | up | 9.89747 | up | -1.69146 | down |
| Stearamide Esi+27.01875 | 4.838047 | up | 4.8526955 | up | 6.897421 | up |
| Oleoyl Ethyl Amide | -3.326086 | down | 6.5634146 | up | 6.31002 | up |
| PC(20:4(5Z,8Z,11Z,14Z)/0:0) Esi+13.5795 | 7.2494087 | up | 7.263236 | up | 7.14277 | up |
| SM(d18:0/14:0) | -0.6475253 | down | 7.0945816 | up | 6.814723 | up |
| C35 H70 N4 O12 | -1.150044 | down | 6.7083035 | up | 6.430974 | up |
| C20 H33 N14 O4 Esi+14.959747 | 7.573964 | up | 6.9822407 | up | 7.023177 | up |
| SM(d18:1/12:0) | 7.0227847 | up | 6.4987235 | up | 6.079561 | up |
| PC(22:6(4E,7E,10E,13E,16E,19E)/0:0)[U] Esi+13.666502 | 7.045806 | up | 7.256194 | up | 6.743042 | up |
| PC(18:2(9Z,12Z)/16:1(9Z)) | 6.673232 | up | 4.7637568 | up | 4.048016 | up |
| PC(18:0/20:4(5Z,8Z,10E,14Z)(12OH[S])) | 6.2216105 | up | 4.6486382 | up | 1.601071 | up |
| Chenodeoxycholic acid glycine conjugate | 8.258264 | up | 8.25221 | up | 8.212152 | up |
| Malyngamide H | 8.046558 | up | 7.822611 | up | 7.68272 | up |
| N-dodecanoyl-L-Homoserine lactone-3-hydrazone-biotin Esi+14.983498 | 7.0731707 | up | 7.2865477 | up | 6.582175 | up |
| 1-(5Z,8Z,11Z,14Z,17Z-Eicosapentaenoyl)-sn-glycero-3-phosphocholine | 6.6068354 | up | 6.3620677 | up | 4.129128 | up |
| PC(O-18:0/O-2:1(1E)) | 6.295921 | up | 5.6280227 | up | 5.300665 | up |
| C20 H29 O | 9.074834 | up | 8.359843 | up | 7.935481 | up |
| LysoPE(0:0/18:2(9Z,12Z)) | 7.670116 | up | 7.280315 | up | 7.104648 | up |
| Metolcarb Esi+0.5825002 | 5.2673006 | up | 6.6362734 | up | 4.356341 | up |
| C17 Sphinganine | 12.870852 | up | 13.046991 | up | 11.53643 | up |
| Campesteryl glucoside | 7.218211 | up | 4.7043495 | up | 4.790616 | up |
| PC(O-16:1(11Z)/2:0) Esi+15.370501 | 6.857312 | up | 6.987922 | up | 6.078271 | up |
| C32 H58 O6 Esi+27.092997 | 2.8257475 | up | 7.5026107 | up | 7.645532 | up |
| Allomaltol | 7.318102 | up | 7.253588 | up | 7.116289 | up |
| PS(O-18:0/18:3(6Z,9Z,12Z)) | 6.00709 | up | 4.3900175 | up | -8.32037 | down |
| C32 H56 O5 Esi+27.044247 | 5.768873 | up | 6.508849 | up | 7.262091 | up |
| (9Z,11E,13E,15Z)-4-Oxo-9,11,13,15-octadecatetraenoic acid | 7.1942654 | up | 7.428114 | up | 7.532505 | up |
| Tridodecylamine | -5.699955 | down | 9.308877 | up | 9.253347 | up |
| LPA(0:0/18:2(9Z,12Z)) | 7.8326044 | up | 7.512091 | up | 4.332737 | up |
| C43 H56 N5 O12 S | 7.509342 | up | 7.789618 | up | 3.31255 | up |
| C28 H52 O5 Esi+26.812496 | 3.30725 | up | 5.3581285 | up | 7.656723 | up |
| SM(d18:1/17:0) | 4.6692677 | up | 9.307497 | up | 2.25357 | up |
| PS(O-18:0/20:3(8Z,11Z,14Z)) Esi+31.797918 | 6.319193 | up | 4.2900653 | up | 0.999805 | up |
| Nonoxynol-9 | 7.1733108 | up | 7.4547367 | up | 2.392423 | up |
| C26 H45 O14 | 7.4288054 | up | 6.9504786 | up | 6.589069 | up |
| Abacavir | 7.83928 | up | 7.5784717 | up | 7.226616 | up |
| C29 H64 N17 O2 | -1.2964482 | down | 7.3099165 | up | 7.029421 | up |
| C38 H83 N4 O14 | 6.485164 | up | 3.2472606 | up | -2.75412 | down |
| PC(20:4(5Z,8Z,11Z,14Z)/18:1(11Z)) 32.58123 | 7.914783 | up | -2.3900476 | down | 11.53661 | up |
| C25 H50 Cl N3 S3 | 6.7540765 | up | 6.3754134 | up | 6.447794 | up |
| PS(O-18:0/22:6(4Z,7Z,10Z,13Z,16Z,19Z)) 30.000254 | 0.2970276 | up | 6.232929 | up | 5.287696 | up |
| C33 H68 N4 O12 | 3.2729573 | up | 6.773448 | up | 6.303093 | up |
| C24 H44 Cl O6 S | 6.614064 | up | 6.6625857 | up | 6.232482 | up |
| C35 H62 N15 O3 | 2.5102139 | up | 6.845443 | up | 7.018756 | up |
| PC(20:5(5Z,8Z,11Z,14Z,17Z)/20:1(11Z)) 35.138985 | -5.8368015 | down | 6.906289 | up | 5.046708 | up |
| Glycerol triundecanoate | 3.5355883 | up | 4.802333 | up | -2.23203 | down |
| LysoPC(22:5(4Z,7Z,10Z,13Z,16Z)) | 6.893742 | up | 6.8991833 | up | 6.176918 | up |
| Chenodeoxycholic acid glycine conjugate Esi+9.022252 | 5.3626842 | up | 7.5145545 | up | 5.641875 | up |
| C28 H52 O5 27.263504 | 4.4779873 | up | 5.858507 | up | 6.623247 | up |
| Diisononyl phthalate Esi+34.07749 | 9.5337515 | up | 9.0458975 | up | 9.121388 | up |
| SM(d18:2/18:1) | 7.018802 | up | 7.1333084 | up | 6.453311 | up |
| C29 H62 N8 O5 S | 7.2062488 | up | -0.5668297 | down | 7.673032 | up |
| C24 H55 N4 O7 | 2.919013 | up | 6.267424 | up | 6.281276 | up |
| Capsi-amide | 5.027504 | up | 7.12885 | up | 6.410975 | up |
| PE-Cer(d14:1(4E)/20:0) | 6.5320024 | up | 6.205397 | up | 5.871933 | up |
| C23 H41 N7 O9 | 7.1789765 | up | 5.374506 | up | 6.426729 | up |
| C27 H39 N7 O9 | 7.499008 | up | 6.7169123 | up | 6.356653 | up |
| HC Toxin | 4.292794 | up | 4.4004517 | up | -6.82347 | down |
| C27 H55 N8 O4 | 6.1978846 | up | -8.083523 | down | 6.276144 | up |
| C21 H43 N8 O | 6.5863495 | up | 6.5430136 | up | 6.700802 | up |
| Trp Arg Met | 7.1868515 | up | 6.7918634 | up | 6.34839 | up |
| C36 H66 O6 | 7.425111 | up | 6.874312 | up | 6.76197 | up |
| Dodecylbenzene | 7.704971 | up | 6.8061724 | up | 6.980717 | up |
| C29 H57 N18 O | 6.3159566 | up | 3.2294016 | up | -2.37261 | down |
| Glycocholic acid | 8.154598 | up | 8.133335 | up | 8.480399 | up |
| C23 H47 N8 O2 Esi+19.535246 | 6.565043 | up | 12.441906 | up | 9.934528 | up |
| LysoPE(0:0/22:6(4Z,7Z,10Z,13Z,16Z,19Z)) | 8.012154 | up | 7.9574547 | up | 7.712368 | up |
| C36 H72 N4 O4 S | 5.1705046 | up | 7.3341193 | up | 6.918259 | up |
| C22 H33 N14 O4 Esi+13.360248 | 7.266905 | up | 6.6198635 | up | 6.026732 | up |
| Ricinoleic Acid methyl ester Esi+14.1735 | 6.8468943 | up | 6.8765492 | up | 6.593623 | up |
| C39 H68 O6 | 6.164626 | up | 0.711607 | up | 2.554313 | up |
| C17 Sphinganine Esi+8.106501 | 7.1128864 | up | 7.751565 | up | 7.517379 | up |
| Ethotoin | 6.9256735 | up | 6.3962574 | up | 6.315824 | up |
| 1-Hexadecylpyridinium | 13.507165 | up | 13.506563 | up | 13.61 | up |
| C19 H36 N7 | 6.4661155 | up | 6.902616 | up | 6.569178 | up |
| Glycidyl stearate | 6.522354 | up | 6.4582047 | up | 6.765244 | up |
| C26 H51 N18 | 6.3535104 | up | 4.8727636 | up | -0.69869 | down |
| C17 Sphinganine Esi+7.608275 | 10.949131 | up | 12.104252 | up | 9.03093 | up |
| C42 H88 N3 O12 | 6.9981956 | up | 5.12134 | up | 4.3073 | up |
| C21 H44 O5 | 7.2124543 | up | 7.1488123 | up | 7.059579 | up |
| C39 H77 N6 O7 S | 6.3521004 | up | 6.0598364 | up | 0.760771 | up |
| PA(12:0/19:0) | 7.666079 | up | 6.9548664 | up | 6.361397 | up |
| C40 H55 O2 | -2.8257847 | down | 6.8210044 | up | 6.171318 | up |
| C35 H68 N3 S2 | 7.686941 | up | 7.1556954 | up | 6.439989 | up |
| Stearamide Esi+24.991747 | 6.0948772 | up | 7.156143 | up | 6.949577 | up |
| PC(15:1(9Z)/0:0) | 6.59218 | up | 6.3577523 | up | 6.820252 | up |
| C23 H37 N6 O11 | 6.6745086 | up | 6.1386666 | up | 6.213123 | up |
| Cyclandelate | 7.0194674 | up | 7.003333 | up | 6.692051 | up |
| C30 H54 O5 | 7.0879335 | up | 7.3088565 | up | 5.876789 | up |
| C24 H42 N6 O14 | 1.0882037 | up | -0.09817505 | down | 5.420153 | up |
| L-Carnitine | 8.047297 | up | 8.262851 | up | 7.73972 | up |
| C44 H83 N4 O12 | 4.741312 | up | 6.604206 | up | 5.939886 | up |
| C15 H32 O7 | 7.092861 | up | 5.431454 | up | 7.379493 | up |
| 1-(9E-Hexadecenoyl)-sn-glycero-3-phosphocholine Esi+13.053996 | 5.9542685 | up | 5.013863 | up | 5.385489 | up |
| PC(20:3(8Z,11Z,14Z)/0:0) Esi+14.708252 | 7.0112305 | up | 6.327962 | up | 6.866041 | up |
| PA(12:0/15:1(9Z)) | 3.621304 | up | 6.712534 | up | 5.680567 | up |
| C22 H45 N O3 | 3.2871974 | up | 6.6172285 | up | 6.564013 | up |
| PS(O-18:0/19:1(9Z)) | 6.221443 | up | 5.020473 | up | -1.90918 | down |
| Hymenoflorin | 7.068569 | up | 7.225585 | up | 4.597273 | up |
| C34 H62 N6 O4 Esi+33.142555 | 8.32273 | up | 7.549943 | up | 8.014895 | up |
| C40 H55 O2 Esi+31.142492 | 2.7031388 | up | 6.855746 | up | 6.566171 | up |
| C27 H43 O6 Esi+18.293243 | 7.2708764 | up | 7.0464864 | up | 6.56494 | up |
| PA(12:0/19:0) Esi+32.59887 | 7.614071 | up | 6.702649 | up | 6.504448 | up |
| C46 H95 N4 O14 | 3.112097 | up | 6.366947 | up | 4.641286 | up |
| C31 H65 N11 O6 | 6.4767065 | up | 3.4357443 | up | -0.44997 | down |
| Trihexosylceramide (d18:1/16:0) | -1.3469958 | down | 6.102585 | up | 5.915065 | up |
| C34 H67 N18 O3 | 6.5578303 | up | 5.156618 | up | -2.20446 | down |
| Glycerophospho-N-Arachidonoyl Ethanolamine | 7.9367313 | up | 8.201426 | up | 7.729691 | up |
| C18-OH Sulfatide | 8.69821 | up | 11.347621 | up | 12.8537 | up |
| C28 H52 O5 Esi+26.211533 | 4.940158 | up | 8.720088 | up | 4.850232 | up |
| C20 H31 Cl O | 6.748528 | up | 6.661338 | up | 6.001492 | up |
| MG(16:0/0:0/0:0) | 7.0181704 | up | 6.2931376 | up | 6.874606 | up |
| PS(O-18:0/18:3(6Z,9Z,12Z)) Esi+30.332254 | 11.387623 | up | 2.6283412 | up | 4.924219 | up |
| C32 H54 N3 O5 | 5.048671 | up | 4.254464 | up | -0.14247 | down |
| Sphingosine | 7.240334 | up | 7.32376 | up | 7.312059 | up |
| C19 H39 N8 | 6.537799 | up | 5.2127247 | up | 6.805292 | up |
| DG(12:0/17:2(9Z,12Z)/0:0)[iso2] | 7.2724805 | up | 5.9363008 | up | 6.142869 | up |
| C26 H41 N O4 S | 10.4479065 | up | 10.74229 | up | 11.7644 | up |
| C30 H56 N3 O5 | 5.4166093 | up | 7.136984 | up | 7.000299 | up |
| CPA(18:1(11Z)/0:0) | 6.749468 | up | 6.8067775 | up | 6.633133 | up |
| C17 H39 N14 O3 | 6.3669806 | up | 4.702948 | up | 7.349752 | up |
| C26 H52 N3 O5 | 6.383634 | up | 6.7581797 | up | 7.288773 | up |
| Nap-Thr-OH | 7.094121 | up | 6.9005637 | up | 5.078676 | up |
| Ricinoleic Acid methyl ester Esi+23.335491 | 6.806295 | up | 7.4323454 | up | 7.840929 | up |
| C19 H35 N8 O7 | 8.687959 | up | 7.8308034 | up | 5.918546 | up |
| SM(d16:1/17:0) 30.989246 | 9.838326 | up | 11.996886 | up | 7.887198 | up |
| Clausarinol | 7.410566 | up | 7.5680513 | up | 7.076744 | up |
| C13 H25 N O2 Esi+26.477997 | 0.35481024 | up | 6.299667 | up | 7.03581 | up |
| Stearoylethanolamide | 6.987694 | up | 7.2399936 | up | 7.160986 | up |
| DG(16:1(9Z)/17:2(9Z,12Z)/0:0)[iso2] | 7.0120354 | up | 6.591352 | up | 6.313268 | up |
| C15 H35 Cl N3 O S | 6.7834835 | up | 4.8250966 | up | 6.430192 | up |
| C33 H64 N11 O7 | 2.908132 | up | 6.9056396 | up | 6.630049 | up |
| C32 H56 O5 Esi+26.187006 | 6.665825 | up | 3.7151875 | up | 7.339785 | up |
| Clausarinol Esi+11.318253 | 5.9358716 | up | 7.5860195 | up | 3.177692 | up |
| C33 H43 O8 S | 6.5132895 | up | 4.6528873 | up | 6.106459 | up |
| C22 H47 Cl N2 O2 | 5.881986 | up | 5.808236 | up | 7.311081 | up |
| Laudanosine | 7.0967627 | up | 3.379329 | up | 4.521276 | up |
| (4E,8E,10E-d18:3)sphingosine | 6.335375 | up | 6.1000614 | up | 6.724011 | up |
| C8 H7 N3 O4 | 6.7153482 | up | 7.1190357 | up | 6.843681 | up |
| C5 H7 N4 O | 6.829844 | up | 7.3936915 | up | 5.974806 | up |
| Tragopogonsaponin M | 6.850281 | up | 5.6160145 | up | 5.062276 | up |
| C32 H56 O5 Esi+26.347744 | 5.5006323 | up | 6.625594 | up | 6.962803 | up |
| Phylloflavan | 6.712554 | up | 6.968256 | up | 2.930866 | up |
| Ser Lys Thr | 6.61404 | up | 3.4463139 | up | 4.705632 | up |
| 1-Benzylimidazole | 6.9705987 | up | 6.3574157 | up | 6.294347 | up |
| DG(12:0/17:2(9Z,12Z)/0:0)[iso2] Esi+28.825129 | 6.6544375 | up | 6.5752945 | up | 6.260011 | up |
| PS(O-18:0/22:6(4Z,7Z,10Z,13Z,16Z,19Z)) Esi+31.737696 | 6.1037984 | up | 1.4231265 | up | 4.700072 | up |
| Creatine | 4.7516713 | up | 7.137691 | up | 7.489883 | up |
| 10-Deoxymethynolide | 7.3333254 | up | 7.4512887 | up | 7.048291 | up |
| C18 H36 N10 O | 7.170682 | up | 5.4832807 | up | 5.821556 | up |
| D-Urobilin | 6.6331515 | up | 4.4756184 | up | 8.291014 | up |
| SM(d18:1/24:1(15Z)) | 4.1245313 | up | 7.3279176 | up | 7.9936 | up |
| PC(18:3(6Z,9Z,12Z)/P-18:1(11Z)) | 7.77931 | up | 14.709634 | up | 11.11858 | up |
| C38 H73 N3 O16 | 2.7979088 | up | 8.230641 | up | 7.862271 | up |
| C18 H34 O3 | 3.358943 | up | 2.288806 | up | 6.03119 | up |
| Phytosphingosine | 5.547593 | up | 8.1878605 | up | 6.044677 | up |
| N-docosahexaenoyl GABA Esi+5.531749 | 8.878938 | up | 8.573125 | up | 8.629648 | up |
| DG(16:1(9Z)/17:1(9Z)/0:0)[iso2] | 10.132732 | up | 6.912081 | up | 0.384204 | up |
| C32 H60 O6 Esi+28.677002 | 10.001947 | up | 9.650604 | up | 7.585569 | up |
| PS(O-20:0/22:6(4Z,7Z,10Z,13Z,16Z,19Z)) Esi+33.674343 | 7.517557 | up | 5.4755154 | up | 2.164912 | up |
| C31 H54 N4 O2 | 3.0879614 | up | 6.7034516 | up | 6.462499 | up |
| C17 Sphinganine Esi+7.412774 | 10.072095 | up | 10.420492 | up | 8.692832 | up |
| Dihydrospheroidene/ Methoxyneurosporene 31.248337 | 2.1206632 | up | 6.94548 | up | 6.407629 | up |
| C34 H54 S | 3.0514927 | up | 5.4660435 | up | 6.375747 | up |
| PS(O-18:0/22:4(7Z,10Z,13Z,16Z)) | 6.479338 | up | 5.200507 | up | -3.60769 | down |
| PA(12:0/19:0) Esi+33.024498 | 9.136705 | up | 8.798642 | up | 7.875508 | up |
| C42 H72 N O5 | 3.3560991 | up | 7.360179 | up | 7.019241 | up |
| Anandamide (20:l, n-9) | 6.6028924 | up | 6.2781034 | up | 6.265382 | up |
| C31 H54 N2 O S2 | 7.128392 | up | 4.90794 | up | 6.435545 | up |
| C19 H35 N4 O3 | 5.892571 | up | 7.2654476 | up | 6.937686 | up |
| Oleoyl Ethyl Amide Esi+18.292246 | 6.7754207 | up | 6.5695815 | up | 6.581897 | up |
| Arapaimol-B | 6.022802 | up | 6.2203326 | up | 7.020715 | up |
| PA(22:6(4Z,7Z,10Z,13Z,16Z,19Z)/0:0) | 9.537122 | up | 5.920128 | up | 5.141614 | up |
| C25 H41 N3 O12 Esi+15.949994 | 6.363863 | up | 6.1204967 | up | 6.375963 | up |
| alpha,4,2'-Trihydroxy-4-O-geranyldihydrochalcone | 7.1945114 | up | 7.3250976 | up | 3.603476 | up |
| C17 H32 N7 O3 | 7.4495645 | up | 5.8120036 | up | 6.136419 | up |
| Gibberellin A9 | 6.7458835 | up | 3.4816656 | up | 4.76102 | up |
| C30 H52 N6 O4 | 7.676034 | up | 9.190012 | up | 9.514052 | up |
| Glucosylceramide (d18:1/16:0) | 5.52015 | up | 10.689552 | up | 7.946592 | up |
| (±)-Octanoylcarnitine | 6.880093 | up | 7.486812 | up | 5.043858 | up |
| Eicosapentaenoic Acid ethyl ester Esi+19.420324 | 5.973179 | up | 6.074247 | up | 6.856295 | up |
| (7R*,8R*)-3-Methoxy-3',4,7,9,9'-pentahydroxy-8,4'-oxyneolignan 4-xyloside | 9.562229 | up | 5.607047 | up | 1.171276 | up |
| C16 H31 N O2 S | 6.894986 | up | 7.2766767 | up | 6.890704 | up |
| C18 H28 | 6.854946 | up | 4.9527006 | up | 6.172601 | up |
| C40 H57 O5 | 3.4271886 | up | 5.337855 | up | 7.207596 | up |
| C33 H66 N10 O7 | 8.012161 | up | 6.5560365 | up | 2.014333 | up |
| Plakortic acid | 8.627107 | up | 9.066989 | up | 7.476741 | up |
| PS(20:3(8Z,11Z,14Z)/18:0) Esi+31.600992 | 16.694775 | up | 10.007603 | up | 10.4188 | up |
| PC(17:1(10Z)/0:0) | 7.123568 | up | 6.7559347 | up | 6.638387 | up |
| C30 H54 N3 O3 | 4.9326057 | up | 6.839678 | up | 6.798746 | up |
| (25S)-5alpha-cholestan-3beta,4beta,6alpha,8beta,15alpha,16beta,26-heptol | 6.7560124 | up | 7.427944 | up | -0.47544 | down |
| PC(14:1(9Z)/19:1(9Z)) | 11.268498 | up | 18.837759 | up | 15.02504 | up |
| C24 H43 N3 O13 | 7.320595 | up | 5.447244 | up | 7.261726 | up |
| Glycidyl stearate Esi+27.695572 | 1.5778666 | up | 3.2460585 | up | 8.467545 | up |
| DG(12:0/17:2(9Z,12Z)/0:0)[iso2] Esi+29.807255 | 5.118758 | up | 6.6882987 | up | 4.957223 | up |
| C13 H25 N O3 | 6.1254816 | up | 6.746829 | up | 6.81976 | up |
| Microlenin | 8.900161 | up | 10.320555 | up | 8.626309 | up |
| CAY10470 Esi+15.819002 | 7.75719 | up | 5.1277246 | up | 6.08171 | up |
| C31 H64 Cl2 N6 | 10.934951 | up | 9.23055 | up | 9.972734 | up |
| Anopterine | 7.165172 | up | 6.7009783 | up | 6.36058 | up |
| PS(20:3(8Z,11Z,14Z)/18:0) Esi+29.98013 | 2.3011913 | up | 5.3449607 | up | 7.029629 | up |
| C18 H34 O3 Esi+32.482254 | 5.8400373 | up | 2.1959758 | up | 10.26759 | up |
| Stearamide Esi+32.947746 | 8.56376 | up | 12.501619 | up | 12.08057 | up |
| C25 H37 N10 O8 | 7.3402233 | up | 7.085388 | up | 6.113211 | up |
| DG(12:0/17:2(9Z,12Z)/0:0)[iso2] Esi+29.207031 | 6.590532 | up | 9.923747 | up | 3.205113 | up |
| C19 H29 N14 O4 | -5.67515 | down | 7.371316 | up | 7.014377 | up |
| LysoPE(0:0/18:2(9Z,12Z)) Esi+13.204251 | 7.6503854 | up | 7.627397 | up | 5.598792 | up |
| C35 H46 Cl O3 S2 | 6.0261636 | up | 5.928417 | up | 6.580377 | up |
| Tiocarbazil | 9.44644 | up | 5.0919476 | up | 9.471853 | up |
| C13 H33 N6 S2 | 5.381879 | up | 7.087521 | up | -0.71172 | down |
| C24:6n-3,5,9,12,16,20 | 6.372254 | up | 8.15967 | up | 5.726308 | up |
| DG(12:0/17:2(9Z,12Z)/0:0)[iso2] Esi+32.087013 | 6.606603 | up | 5.16503 | up | 3.096506 | up |
| C36 H33 N8 O4 | 7.027322 | up | 5.690354 | up | 5.663247 | up |
| 1,2,10-Trihydroxydihydro-trans-linalyl oxide 7-O-beta-D-glucopyranoside | 6.924359 | up | 4.16769 | up | 4.416905 | up |
| PA(12:0/19:0) Esi+32.424507 | 7.22939 | up | 6.923474 | up | 5.609545 | up |
| 1-Octene | -6.7426696 | down | 6.799791 | up | 6.842444 | up |
| C21 H50 N12 O2 S | 7.6106095 | up | 7.7278852 | up | 7.926726 | up |
| C27 H50 O3 | 6.1435432 | up | 5.5275908 | up | 7.505398 | up |
| C37 H33 N4 O4 | 5.188206 | up | 6.5416293 | up | 7.889123 | up |
| dodecanamide | 6.236771 | up | 7.0099087 | up | 5.613094 | up |
| C21 H46 N7 O3 | 6.0052495 | up | 2.679266 | up | 8.087528 | up |
| Glycerol triundecanoate Esi+32.79502 | 9.352177 | up | 6.294915 | up | 6.431561 | up |
| C30 H45 N3 O7 | 3.305853 | up | 6.4633737 | up | 6.445925 | up |
| C20 H33 N14 O4 Esi+15.005249 | 7.9535255 | up | 7.269939 | up | 7.031931 | up |
| DG(12:0/17:2(9Z,12Z)/0:0)[iso2] Esi+31.128101 | 6.726391 | up | 7.1079984 | up | 6.472137 | up |
| C33 H33 N3 O2 | 11.853144 | up | 9.222469 | up | 10.26298 | up |
| C16:4n-0,4,8,12 | 7.041172 | up | 7.1523533 | up | 7.433627 | up |
| C30 H28 Cl N4 O4 | 6.646536 | up | 6.5871677 | up | 6.867724 | up |
| C40 H86 N6 O15 | 8.98776 | up | 5.5545654 | up | -0.49051 | down |
| Anandamide (20:l, n-9) Esi+24.360752 | 7.3002253 | up | 7.210786 | up | 7.439963 | up |
| PC(18:3(6Z,9Z,12Z)/0:0)[U] Esi+15.032251 | 7.599864 | up | 6.6268144 | up | 6.566607 | up |
| Ile Arg Pro | 7.341709 | up | 7.868294 | up | 6.747656 | up |
| Minabeolide-8 | 8.613811 | up | 5.288019 | up | 7.241904 | up |
| Docosanamide Esi+32.47776 | -1.9143353 | down | 6.7081833 | up | 6.321279 | up |
| Ganoderic acid V | 7.209771 | up | 5.4218464 | up | 6.203297 | up |
| TG(8:0/8:0/8:0) | 7.674679 | up | 7.191433 | up | 4.023507 | up |
| C25 H54 N3 O7 | 7.427255 | up | 7.3192487 | up | 6.957614 | up |
| C22 H41 N10 O10 | 4.216712 | up | 6.8821917 | up | 4.807017 | up |
| 1,3-Octadiene | 5.6346087 | up | 5.502273 | up | 7.525529 | up |
| Propapyriogenin A2 | 7.4918523 | up | 5.5440426 | up | 6.368088 | up |
| C29 H58 N10 O6 | 7.1887703 | up | 5.6514096 | up | -0.8268 | down |
| C28 H41 N6 O6 S2 | 10.151222 | up | 10.334699 | up | -3.15321 | down |
| C13 H13 N5 O3 S | 7.353182 | up | 6.454749 | up | 4.677167 | up |
| C17 Sphinganine Esi+8.76725 | 7.00748 | up | 6.740701 | up | 6.617211 | up |
| LysoPE(0:0/22:6(4Z,7Z,10Z,13Z,16Z,19Z)) Esi+13.533751 | 7.9455924 | up | 8.238814 | up | 7.615543 | up |
| PC(16:0/22:6(4E,7E,10E,13E,16E,19E)) Esi+27.589 | 7.721433 | up | 6.4826946 | up | -0.1724 | down |
| Asp Lys Pro | 6.5471644 | up | 4.2488565 | up | 7.159879 | up |
| 1-O-alpha-D-glucopyranosyl-1,2-eicosandiol | 6.975314 | up | 6.789056 | up | 6.632125 | up |
| (2S,4R,6R)-2-Methyl-6-nonylpiperidin-4-ol | 6.7614894 | up | 6.8148937 | up | 6.762012 | up |
| C36 H63 N20 O | 9.820894 | up | 13.845317 | up | 11.44605 | up |
| JWH145 | 3.6697083 | up | 5.079953 | up | 6.802587 | up |
| Campesteryl glucoside Esi+28.6704 | 10.929713 | up | 7.7638326 | up | 0.611221 | up |
| Citranaxanthin | 7.2078094 | up | 8.024223 | up | 8.155413 | up |
| DG(16:1(9Z)/17:1(9Z)/0:0)[iso2] Esi+32.511997 | 7.5576944 | up | 6.834395 | up | 6.644503 | up |
| C22 H43 Cl N8 O | 2.6574025 | up | 6.851907 | up | 6.468498 | up |
| C24:6n-3,5,9,12,16,20 9.56 | 4.0705266 | up | 6.744139 | up | 4.804013 | up |
| Glycerophospho-N-Arachidonoyl Ethanolamine Esi+13.443502 | 7.434878 | up | 7.7692733 | up | 7.480272 | up |
| C20 H26 Esi+16.628756 | 4.547497 | up | 1.8525691 | up | 6.151952 | up |
| C39 H47 N O2 | 5.624004 | up | 6.560258 | up | 7.630602 | up |
| C49 H99 N4 O10 S2 | 4.0878186 | up | 6.9248753 | up | 7.271061 | up |
| PA(18:1(9Z)/13:0) | 7.1844254 | up | 7.0892687 | up | 6.357491 | up |
| Dextrothyroxine | 7.160679 | up | 6.776811 | up | 6.37747 | up |
| N-Oleoyl-L-Serine | 8.27393 | up | 8.036178 | up | 7.9538 | up |
| LysoPE(0:0/22:5(4Z,7Z,10Z,13Z,16Z)) | 7.630895 | up | 7.337297 | up | 7.201387 | up |
| C33 H62 O S2 | 7.1099114 | up | 6.680182 | up | 7.499198 | up |
| PC(O-1:0/O-16:0)[U] Esi+17.175253 | 6.290147 | up | 3.0115252 | up | 10.66445 | up |
| C30 H67 N19 O6 | 6.8550696 | up | 4.980303 | up | 7.176673 | up |
| Capsi-amide Esi+24.124994 | 6.469774 | up | 1.8748074 | up | 6.964016 | up |
| C31 H62 N10 O6 | 7.4444337 | up | 5.273835 | up | -0.44919 | down |
| C32 H58 O6 Esi+27.071747 | 4.0606165 | up | 5.9965825 | up | 8.290785 | up |
| Trp Arg Met Esi+14.1742525 | 0.98071384 | up | 5.616633 | up | 6.575919 | up |
| C23 H44 N7 O5 | 7.1779866 | up | 2.7234826 | up | 6.435804 | up |
| zymosterol intermediate 1c | 5.765486 | up | 9.270956 | up | 8.177576 | up |
| C30 H54 O5 Esi+28.87923 | 1.7516031 | up | 6.437657 | up | 6.305232 | up |
| C18 H39 N7 O8 | 8.512745 | up | 10.072031 | up | 10.97528 | up |
| PA(20:3(8Z,11Z,14Z)/0:0) | 7.483752 | up | 6.9523916 | up | 5.264971 | up |
| C36 H47 Cl2 O | 7.2657976 | up | 6.987396 | up | 6.780271 | up |
| C8 H7 N3 O4 Esi+0.5297498 | 7.21695 | up | 7.3944187 | up | 7.075361 | up |
| Momordol | 7.6742587 | up | 6.5387506 | up | 6.719468 | up |
| N-docosahexaenoyl GABA Esi+5.3490005 | 9.191163 | up | 9.743983 | up | 9.615934 | up |
| PA(18:1(9Z)/13:0) Esi+30.927753 | 7.448318 | up | 7.0863767 | up | 6.739557 | up |
| C23 H46 N10 O5 | 6.599143 | up | 5.2925205 | up | 1.176883 | up |
| Cannabidiol dimethyl ether | 1.2205791 | up | 11.549028 | up | 10.55707 | up |
| C20 H32 Cl N17 O3 | 7.323138 | up | 6.1317635 | up | 6.20408 | up |
| Stigmast-4-ene-3,6-dione | 8.563525 | up | 5.2475643 | up | 4.666995 | up |
| C42 H83 N18 O7 | 6.895952 | up | 3.305414 | up | -2.5933 | down |
| C27 H48 N5 S2 | 3.4282835 | up | -1.2768364 | down | 7.08136 | up |
| L-759,633 | 8.082936 | up | 9.093002 | up | 8.325406 | up |
| PS(P-16:0/12:0) | 12.50997 | up | 14.982954 | up | 12.06058 | up |
| C30 H57 Cl N5 O | 4.736025 | up | 6.1093564 | up | 7.686698 | up |
| C23 H41 N7 O9 Esi+15.374748 | 7.4042244 | up | 6.7214313 | up | 6.645134 | up |
| C34 H61 N O | 1.7275186 | up | 8.859309 | up | 8.540386 | up |
| Neolinderatin | 4.959091 | up | 7.105812 | up | 6.486001 | up |
| L-a-Lysophosphatidylserine | 4.04762 | up | 5.646048 | up | -3.51164 | down |
| Longifolonine Esi+3.7177498 | 2.552805 | up | 5.808696 | up | 6.692945 | up |
| C10 H10 | 4.9075427 | up | 3.8692865 | up | 6.556757 | up |
| Oleoyl Ethanolamide | 7.9902964 | up | 7.9916396 | up | 8.342235 | up |
| C23 H45 N4 O5 | 6.0739255 | up | 7.883777 | up | 7.65058 | up |
| Nevskin | 6.114424 | up | 7.7739954 | up | 7.924871 | up |
| Heptadecanoyl Ethanolamide | 5.0313687 | up | 3.1853988 | up | 6.41695 | up |
| 1-Palmitoyl Lysophosphatidic Acid | 11.413478 | up | 6.139524 | up | 8.10593 | up |
| C35 H36 Cl N O5 | 6.4959917 | up | 6.7026973 | up | 6.486746 | up |
| C30 H60 N2 O3 S2 | 6.0844526 | up | 4.880232 | up | 6.740225 | up |
| C16 H40 N6 S2 | 8.4459505 | up | 7.0653715 | up | 1.774404 | up |
| PI(20:4(5Z,8Z,11Z,14Z)/0:0) Esi+13.8495 | 6.653512 | up | 7.171315 | up | 6.452447 | up |
| Benzoquinoneacetic acid | 3.8095436 | up | 6.5774198 | up | 6.957374 | up |
| Phorbol myristate acetate | 6.825825 | up | 2.6626923 | up | 6.420213 | up |
| Bilirubin | 10.271159 | up | 10.577792 | up | 10.24972 | up |
| Nalorphine | 0.86977625 | up | 5.9692655 | up | -0.96307 | down |
| Acutilol A | 6.6912813 | up | 4.551317 | up | 4.984583 | up |
| Eicosapentaenoic acid-d5 | 6.8137217 | up | 7.3632293 | up | 7.186508 | up |
| Clausarinol Esi+10.518747 | 7.637424 | up | 8.672125 | up | 8.366356 | up |
| PA(16:0/0:0)[cyclic] Esi+18.476757 | 7.904615 | up | 5.4489326 | up | 10.40576 | up |
| C24 H32 O | 7.6540294 | up | 7.8963866 | up | 7.311447 | up |
| N-docosahexaenoyl GABA Esi+5.8254995 | 8.712199 | up | 8.808106 | up | 8.648638 | up |
| N-methylundec-10-enamide | 6.8419065 | up | 5.2209435 | up | 6.833944 | up |
| Allomaltol Esi+7.035249 | 7.152215 | up | 7.3806567 | up | 7.132112 | up |
| PC(P-17:0/0:0) | 6.1263165 | up | 5.9202733 | up | 0.397071 | up |
| LysoPE(0:0/20:3(11Z,14Z,17Z)) | 7.6457424 | up | 7.257986 | up | 5.77153 | up |
| PC(16:0/O-1:0) Esi+16.430996 | 6.9048777 | up | 6.205705 | up | 9.674372 | up |
| 1-(5Z,8Z,11Z,14Z,17Z-Eicosapentaenoyl)-sn-glycero-3-phosphocholine Esi+9.329504 | 6.5588803 | up | 6.266022 | up | 3.068261 | up |
| C31 H50 N4 O | 4.667593 | up | 6.2724576 | up | 3.679912 | up |
| LysoPE(0:0/22:5(4Z,7Z,10Z,13Z,16Z)) Esi+14.7599535 | 7.5700884 | up | 7.5839386 | up | 4.578125 | up |
| C37 H75 N7 S2 | 3.6509385 | up | 7.0570297 | up | 5.029843 | up |
| C25 H37 N10 O8 Esi+14.105499 | 10.152859 | up | 17.003082 | up | 12.78339 | up |
| (25S)-5alpha-cholestan-3beta,6alpha,7beta,8beta,15alpha,16beta,26-heptol | 8.025669 | up | 7.7017384 | up | 6.376899 | up |
| Etonogestrel Esi+10.485 | 10.443431 | up | 9.817691 | up | 8.606759 | up |
| Tiliacorine | 7.745036 | up | 7.5801425 | up | 8.081931 | up |
| (9Z,11E,13E,15Z)-4-Oxo-9,11,13,15-octadecatetraenoic acid Esi+18.971994 | 6.5825896 | up | 6.925185 | up | 6.935841 | up |
| Dihydroisoalantolactone Esi+23.932003 | 6.960415 | up | 7.278235 | up | 6.621233 | up |
| Theasapogenol A | 7.921896 | up | 10.144044 | up | 10.42553 | up |
| (3b,6b,8b,12a)-8,12-Epoxy-7(11)-eremophilene-6-angeloyloxy-8,12-dimethoxy-3-ol | 9.765829 | up | 9.787137 | up | 9.971243 | up |
| (6E,8E)-4,6,8-Megastigmatriene | 8.3686905 | up | 7.4099956 | up | 7.496685 | up |
| C37 H42 Cl N2 O3 | 7.0755563 | up | 7.1014504 | up | 7.611121 | up |
| C22 H35 N14 O4 Esi+15.947124 | 7.804771 | up | 7.2037377 | up | 6.703209 | up |
| L-Urobilinogen | 8.295292 | up | 9.036245 | up | 8.152837 | up |
| N-docosahexaenoyl GABA Esi+6.0555024 | 8.041949 | up | 8.692999 | up | 9.398069 | up |
| PC(17:1(10Z)/0:0) Esi+26.582605 | 5.7833986 | up | 7.517753 | up | 7.02674 | up |
| Typhasterol | 4.3525243 | up | 6.034662 | up | 7.257076 | up |
| PA(O-16:0/14:0) | 3.701105 | up | 6.690384 | up | 7.008751 | up |
| C20 H28 | 7.273512 | up | 5.1612244 | up | 7.114933 | up |
| Allomaltol Esi+5.135751 | 5.234894 | up | 5.582447 | up | 8.100851 | up |
| PA(22:6(4Z,7Z,10Z,13Z,16Z,19Z)/0:0) Esi+16.243505 | 7.8444986 | up | 6.2042403 | up | 4.363647 | up |
| (3a,5b,7b)-24-[(carboxymethyl)amino]-7-hydroxy-24-oxocholan-3-yl-b-D-glucopyranosiduronic acid, | 9.029079 | up | 10.095432 | up | 8.765924 | up |
| Eicosapentaenoic acid-d5 Esi+10.727402 | 7.156834 | up | 5.723192 | up | 7.619674 | up |
| (3beta,5alpha,6beta,22E,24R)-23-Methylergosta-7,22-diene-3,5,6-triol | 3.7736254 | up | 7.147147 | up | 6.96201 | up |
| Lys Phe Phe | 6.335244 | up | 7.952508 | up | 5.835177 | up |
| Pyrohyperforin Esi+31.297842 | 5.300683 | up | 6.692659 | up | 6.745119 | up |
| DMG-MINO | 6.93959 | up | 5.518151 | up | 6.602031 | up |
| C26 H50 N3 O5 | 6.2817554 | up | 6.3070073 | up | 7.198943 | up |
| Dihydrospheroidene/ Methoxyneurosporene Esi+31.529552 | 8.678199 | up | 4.6788297 | up | 8.119801 | up |
| Spheroidene | 7.15156 | up | 7.061389 | up | 7.288038 | up |
| Anandamide (22:6, n-3) | 7.545705 | up | 7.769453 | up | 6.877688 | up |
| Theasapogenol A Esi+21.711496 | 11.106906 | up | 11.547382 | up | 10.82286 | up |
| C28 H23 Cl N O5 | 7.884222 | up | 7.778832 | up | 3.678203 | up |
| PGF2α isopropyl ester | 8.003516 | up | 8.574128 | up | 4.994031 | up |
| C23 H54 N5 S3 | 6.6132054 | up | 7.1422143 | up | 6.863342 | up |
| C26 H54 Cl N6 O | 4.055297 | up | 6.783316 | up | 8.51734 | up |
| Prosopinine | 9.637955 | up | 9.087069 | up | 6.995776 | up |
| 1,3-Diisopropylbenzene Esi+23.151745 | 9.759898 | up | 9.133932 | up | 8.734418 | up |
| Linoleoyl Ethanolamide | 7.342802 | up | 7.233367 | up | 7.40326 | up |
| (4-Hydroxybenzoyl)choline | 11.094201 | up | 10.664075 | up | 8.467453 | up |
| Typhasterol Esi+29.642752 | -1.5725255 | down | 5.238031 | up | 6.210806 | up |
| C31 H62 O S2 | 8.751976 | up | 1.8716059 | up | 9.04099 | up |
| Choline | 8.377913 | up | 8.779622 | up | 8.318548 | up |
| C35 H36 Cl N O5 Esi+14.913504 | 6.747523 | up | 6.6612444 | up | 7.145742 | up |
| Diphenoxylate | -4.291382 | down | 3.4259787 | up | 5.205918 | up |
| C27 H52 O3 | 2.8358989 | up | 8.569144 | up | 5.859532 | up |
| Momordol Esi+25.263502 | 7.9807096 | up | 7.275447 | up | 8.004634 | up |
| Hexacosanedioic acid | 7.29267 | up | 7.27562 | up | 6.832233 | up |
| C18 H26 O3 | 7.087365 | up | 8.108208 | up | 6.377796 | up |
| Malyngamide H Esi+5.8844986 | 4.9466066 | up | 8.487902 | up | 3.802006 | up |
| N-Oleoyl-L-Serine Esi+10.226499 | 9.743162 | up | 7.5150394 | up | 8.874962 | up |
| DG(16:1(9Z)/17:1(9Z)/0:0)[iso2] Esi+32.906746 | 7.247077 | up | 6.9878345 | up | 6.267473 | up |
| Theasapogenol A Esi+22.244747 | 7.971543 | up | 9.142878 | up | 9.427624 | up |
| Silafluofen | 5.219717 | up | 8.912352 | up | 7.323397 | up |
| C12 H14 | 7.820123 | up | 7.629219 | up | 7.403415 | up |
| Niazimicin A | -2.4094129 | down | 5.4435277 | up | 5.498907 | up |
| Palmitoyl Ethanolamide-d4 | 7.5565896 | up | 7.437677 | up | 7.037559 | up |
| Sapienic acid | 7.3893776 | up | -6.407637 | down | 8.259213 | up |
| Trp Trp Gln | -0.5286617 | down | 8.468646 | up | 8.780328 | up |
| (3beta,5alpha,6beta,22E,24R)-23-Methylergosta-7,22-diene-3,5,6-triol 27.030582 | 5.3421116 | up | 5.308395 | up | 10.49435 | up |
| (4Z,7Z,10Z,13Z,16Z,19Z)-Docosahexaenoic acid ethyl ester | 13.749927 | up | 13.930845 | up | 11.77873 | up |
| Amdinocillin | 7.156009 | up | 6.3079557 | up | 8.09782 | up |
| 9Z-Pentatriacontene | -2.1641245 | down | 9.11068 | up | 9.030176 | up |
| PA(12:0/15:1(9Z)) Esi+27.102488 | 9.18845 | up | 9.29521 | up | 10.99531 | up |
| DG(16:1(9Z)/17:1(9Z)/0:0)[iso2] Esi+32.769005 | 8.025022 | up | 6.91194 | up | 6.647419 | up |
| PA(12:0/13:0) | 9.629331 | up | 7.424677 | up | 7.059454 | up |
| DG(16:1(9Z)/17:1(9Z)/0:0)[iso2] Esi+32.244377 | 7.0522976 | up | 6.5759406 | up | 6.458229 | up |
| Norketotifen | 5.9775 | up | 8.996396 | up | 10.18986 | up |
| C20 H42 N4 O20 | 14.422147 | up | 12.598575 | up | 15.85315 | up |
| 10-Deoxymethynolide Esi+3.5520012 | 7.3880386 | up | 6.8368797 | up | 5.863798 | up |
| N-docosahexaenoyl GABA Esi+4.858749 | 6.53927 | up | 8.298773 | up | 12.33827 | up |
| DG(16:1(9Z)/17:2(9Z,12Z)/0:0)[iso2] Esi+30.931751 | 6.8149805 | up | 6.6770186 | up | 6.073564 | up |
| N-Cyclohexanecarbonylpentadecylamine Esi+30.882746 | -6.8726044 | down | 12.180127 | up | 12.03642 | up |
| SM(d18:0/16:0) 34.2825 | 6.7804217 | up | 25.390562 | up | 4.700008 | up |
| Stearamide Esi+26.37801 | 6.5118814 | up | 6.630607 | up | 8.687534 | up |
| PS(O-18:0/22:6(4Z,7Z,10Z,13Z,16Z,19Z)) Esi+31.414951 | 14.46026 | up | 11.53677 | up | 5.659889 | up |
| C27 H55 N13 O | 1.0216298 | up | 6.5309477 | up | 8.25107 | up |
| C51 H30 Cl N7 | 4.4928675 | up | 15.188616 | up | 10.47375 | up |
| PC(18:3(6Z,9Z,12Z)/P-18:1(11Z)) Esi+31.679749 | 19.097618 | up | 8.120831 | up | 5.276556 | up |
| PE-Cer(d15:1(4E)/22:0(2OH)) | 1.6070671 | up | 11.11105 | up | 7.683557 | up |
| Arapaimol-B Esi+32.77492 | 5.635848 | up | 9.345965 | up | 9.290689 | up |
| PS(O-20:0/22:6(4Z,7Z,10Z,13Z,16Z,19Z)) 33.173264 | 1.3593721 | up | 12.248175 | up | 9.838415 | up |
| C25 H46 N4 | 9.663356 | up | 12.910448 | up | 13.02818 | up |
| C21 H43 N8 O Esi+19.561996 | 3.772645 | up | 9.305388 | up | 9.405834 | up |
| C45 H66 N2 O4 | 9.693211 | up | 9.715147 | up | 11.71978 | up |
| C29 H52 N O2 | 1.8820992 | up | 15.113227 | up | 8.429789 | up |
| Cer(d14:1(4E)/24:0(2OH)) | 8.369402 | up | 19.332907 | up | 4.777077 | up |
| C30 H17 N8 O25 S | 13.332281 | up | 13.302464 | up | 15.00817 | up |
| C34 H56 S | 8.136785 | up | 8.548094 | up | 8.046082 | up |
| C23 H28 N O3 S3 | 7.0808406 | up | 6.669243 | up | 10.65024 | up |
| Metolcarb Esi+1.00225 | 7.5304027 | up | 3.4202495 | up | 9.702456 | up |
| C34 H62 N6 O4 Esi+34.545383 | 10.41455 | up | 5.427345 | up | 9.720872 | up |
| Camptothecin | 6.4350295 | up | 7.584145 | up | 11.67562 | up |
| Betaine aldehyde | 16.890059 | up | 16.373043 | up | 16.11678 | up |
| Etonogestrel Esi+11.697248 | 9.800243 | up | 9.884239 | up | 11.34763 | up |
| C31 H62 N S | 4.9674606 | up | 14.700054 | up | 11.4119 | up |
| MIPC(d18:0/26:0(2OH)) | 6.7485623 | up | 9.83916 | up | 9.917434 | up |
| C36 H52 S | 12.80183 | up | 9.921194 | up | 7.665241 | up |
| Isomigrastatin | 0.8670769 | up | 10.093675 | up | 10.02813 | up |
| C27 H47 N3 O2 | 7.1184745 | up | 11.651213 | up | 12.3667 | up |
| Tauroursodeoxycholic acid | 11.092313 | up | 11.255123 | up | 12.90875 | up |
| (5-Heptyl-6-methyloctahydroindolizin-8-yl)methanol 30.928753 | 3.4845514 | up | 6.628762 | up | 13.28848 | up |
| (2S,4R,6R)-2-Methyl-6-nonylpiperidin-4-ol Esi+18.546001 | 8.226641 | up | 10.0289345 | up | 10.93475 | up |
| Nerolidol | 1.9724884 | up | 5.0384207 | up | 13.09815 | up |
| Indoprofen | 7.8929043 | up | 14.134775 | up | 14.29746 | up |
| C29 H50 N7 | 19.852816 | up | 19.829687 | up | 19.53586 | up |
| Glaucarubin | 8.834824 | up | 9.553717 | up | 9.491243 | up |
| Plakortic acid Esi+21.912506 | 9.450161 | up | 4.17905 | up | 9.504476 | up |
| Murrayazolinol | 13.541897 | up | 13.660939 | up | 6.486813 | up |
| PE(17:2(9Z,12Z)/0:0) | 16.0769 | up | 19.121218 | up | 17.38105 | up |
| Melleolide M | 11.369193 | up | 10.333943 | up | 7.050162 | up |
| 9a-Fluorotetrahydrocortisol | 8.251734 | up | 9.51585 | up | 9.509136 | up |
| D1-2-Hydroxymethylethisterone | 6.296053 | up | 3.21924 | up | -3.5999 | down |
| Kurilensoside H | 12.439947 | up | 12.77146 | up | 12.06088 | up |
| PS(18:4(6Z,9Z,12Z,15Z)/0:0) | 8.502907 | up | 6.8132887 | up | 6.507063 | up |
| C32 H59 Cl N2 O2 | 13.60981 | up | 9.913202 | up | 15.72675 | up |
| Oryzarol | 9.648646 | up | 10.352592 | up | 9.918128 | up |
| 1,2-Di-(9Z,12Z,15Z-octadecatrienoyl)-3-(Galactosyl-alpha-1-6-Galactosyl-beta-1)-glycerol | 11.678074 | up | 11.851952 | up | 9.13312 | up |
| Labda-7,14-dien-6-one, 13-hydroxy 18.625246 | 7.87333 | up | 6.0285125 | up | 12.38017 | up |
| Progesterone | 5.46638 | up | 5.477268 | up | 14.08645 | up |
| Chamuvaritin Esi+11.290754 | 12.765093 | up | 11.301662 | up | 6.838357 | up |
| Dimethisterone 20.800497 | 13.239623 | up | 14.59421 | up | 12.51298 | up |
| Dodecylbenzene 27.024496 | 2.1240563 | up | 7.617139 | up | 9.400866 | up |
| (S)-Rutaretin | 6.6977854 | up | 10.738012 | up | 5.525951 | up |
| D1-2-Hydroxymethylethisterone Esi+5.4679995 | 13.955603 | up | 13.62591 | up | 13.38863 | up |
| Phenolic steroid | 7.6935873 | up | 5.0720506 | up | 7.638272 | up |
| Armillaricin | 10.061077 | up | 12.552437 | up | 5.44232 | up |
| Nap-Thr-OH Esi+8.430746 | 12.166016 | up | 13.560048 | up | 5.8392 | up |
| C35 H35 O4 S | 13.363295 | up | 15.529034 | up | 13.92765 | up |
| Gln Pro Gln | 9.014299 | up | 7.306492 | up | 5.647453 | up |
| γ-Linolenic Acid Esi+14.073748 | 11.780894 | up | 4.1423817 | up | 7.42857 | up |
| Octadecyl fumarate | 16.628918 | up | 9.674337 | up | 9.718885 | up |
| C30 H56 N O9 S | 6.1368184 | up | 4.1985426 | up | 15.84515 | up |
