# Supplementary figures and images for: Correlation of host inflammatory cytokines and immune-related metabolites, but not viral NS1 protein, with disease severity of dengue virus infection
Source: PLoS One. 2020 Aug 7;15(8):e0237141. doi: 10.1371/journal.pone.0237141 (PMC7413495; doi:10.1371/journal.pone.0237141)

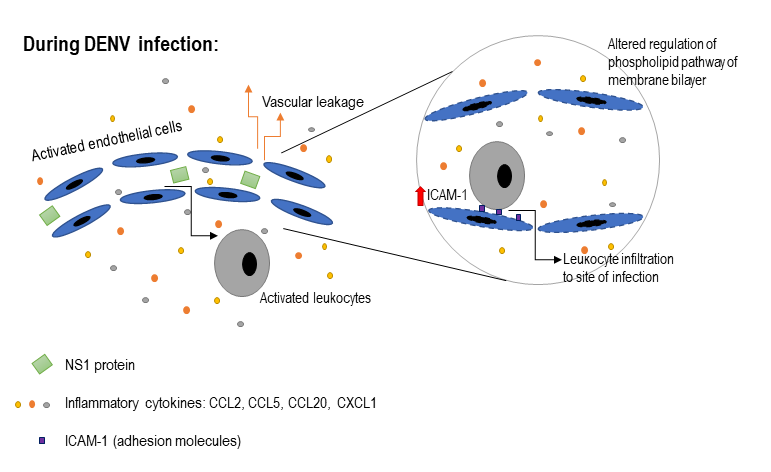

Supplement: S1 Fig — (TIF) [file pone.0237141.s002.tif]
