# Supplementary material for: Correlation of host inflammatory cytokines and immune-related metabolites, but not viral NS1 protein, with disease severity of dengue virus infection
Source: PLoS One. 2020 Aug 7;15(8):e0237141. doi: 10.1371/journal.pone.0237141 (PMC7413495; doi:10.1371/journal.pone.0237141)
Supplement: Attachment — Submitted filename: Response to Reviewers.docx [file pone.0237141.s003.docx]

**Response to Reviewers:**

Reviewers' comments:

Reviewer's Responses to Questions

**Comments to the Author**

1. Is the manuscript technically sound, and do the data support the conclusions?

The manuscript must describe a technically sound piece of scientific research with data that supports the conclusions. Experiments must have been conducted rigorously, with appropriate controls, replication, and sample sizes. The conclusions must be drawn appropriately based on the data presented.

Reviewer #1: Yes

***Response: Thank you.***

2. Has the statistical analysis been performed appropriately and rigorously?

Reviewer #1: I Don't Know

***Response: For cytokine profiling, the precision and reproducibility of the assays were assured by calculating the coefficient of variance of the replicates. Then we proceeded to access the significant differences of the cytokine expression profile between the categories using student’s T test. For untargeted metabolomics, all the statistical analysis was performed using the chemometrics software Mass Profiler Professional designed for the such purposes.***

3. Have the authors made all data underlying the findings in their manuscript fully available?

The [PLOS Data policy](http://www.plosone.org/static/policies.action#sharing) requires authors to make all data underlying the findings described in their manuscript fully available without restriction, with rare exception (please refer to the Data Availability Statement in the manuscript PDF file). The data should be provided as part of the manuscript or its supporting information, or deposited to a public repository. For example, in addition to summary statistics, the data points behind means, medians and variance measures should be available. If there are restrictions on publicly sharing data—e.g. participant privacy or use of data from a third party—those must be specified.

Reviewer #1: No

***Response: The raw data of untargeted metabolomics have been prepared in full in supplementary S1 table (at end of manuscript).***

 4. Is the manuscript presented in an intelligible fashion and written in standard English?

PLOS ONE does not copyedit accepted manuscripts, so the language in submitted articles must be clear, correct, and unambiguous. Any typographical or grammatical errors should be corrected at revision, so please note any specific errors here.

Reviewer #1: Yes

***Response: Thank you.***

5. Review Comments to the Author

Please use the space provided to explain your answers to the questions above. You may also include additional comments for the author, including concerns about dual publication, research ethics, or publication ethics. (Please upload your review as an attachment if it exceeds 20,000 characters)

Reviewer #1: This study by Soe and collegues presents a thorough characterization of the effect of patients’ sera with a broad range of disease severity, or NS1 antigen itself, on the integrity of microvascular endothelial cells. In the attempt of clarifying the ongoing debate on the role of NS1 in disrupting functional endothelial barrier, and its causal relation with Dengue disease progression, the authors further profiled the level of several cytokines and multiple metabolites using a multiplex flow cytometric assay and untargeted metabolomics, respectively. Overall the study is clearly written, results presented in an intelligible fashion and discussed in the context of the vast body of literature on the topic. Although no major breakthrough is presented, and most of the results are actually supportive of a lack of correlation between Dengue disease severity and any of the analysed variables (NS1 levels, pro-inflammatory cytokines or metabolites), the presented results are still important and worth publishing as they consolidate the notion that no specific/individual factor present in the sera (either of viral or cellular origin) can be clearly and unequivocally used as prognostic marker for disease severity. Furthermore, the study present a metabolic-based survey of patients sera, which have been well-characterized/normalized (i.e. based on NS1 levels and IgG), and therefore could be of relevance for the community.

Major points:
My only experimental concern relates to the ECIS experiment, with respect to the control used and the degree of details provided in the methods section.
More specifically (related to Fig.1-3):
- The purified NS1 protein used in Fig.1 was of which origin (i.e. bacterial, mammalian..etc), to which extent was purified, and in which diluent was diluted? This information is quite critical as it sets the ground for interpretation of the assay in the following figures.

***Response: The purified NS1 protein is a recombinant dengue virus serotype 1 NS1 protein of sequence strain Nauru/Western Pacific/1974 expressed in 293 human cells. It is a purified recombinant protein with a C-terminal 6x His-tag and purity of >95% by SDS PAGE analysis diluted in buffer solution Dulbecco’s phosphate buffered saline, which was further diluted during the experiment using the respective media of each cell line used. The above information has been added to the methodology.***

Furthermore, in order to exclude additional factors, the purified NS1 protein used here should have been diluted in healthy individual sera (perhaps this information is provided, though I could not find it in the text or the methods section).

***Response: Healthy individual served as negative control for the experiment and hence it contains no NS1 antigen. It was included to compare against the patients from the three categories of dengue. On the other hand, the purified NS1 protein was included not only to investigate the effect of viral factor (NS1) on endothelium, but also as a comparison for the different NS1 level present in the serum samples. The characteristics of all samples were included in table 1.***

- What are the positive ctrls used in this assay? (probably a known disruptor of endothelial barrier should be included here to show the sensitivity of the assay)

***Response: A positive control was not included in the experiment as a standard disruptor of the endothelial barrier is yet to be established. We therefore included the purified NS1 protein to compare the modulation of endothelial barrier against patient sera that contain NS1 antigen at varying level with the presence of other host factors.***

- What is the dynamic range of this assay? Without the ctrls described above, is difficult to assess whether the amplitude of the changes described in Fig. 1-3 is significant, and whether the variance across and within groups is acceptable.

***Response: The use of ECIS system allow us to monitor real-time cell barrier function, cell to cell adhesion and cell to matrix interactions, which is an incredibly sensitive (described below) method for evaluating barrier function of cells in vitro. It was used to compare the relative pattern of modulation between treated and untreated cells, rather than an absolute reading on the impedance changes. The results presented in this manuscript have been normalized and standardized to eliminate the effect of any gross influences during the experiment. The values were detected at multiple frequencies for three biological replicates with 10 detection points each, and the standard error of the mean was plotted as error bar in Fig. 1-3. The statistical measures included the normalization to a cell-free electrode, standardization of the fluctuations due to cell mobility such as micromotion, and a mathematical model that was implied to determine the changes in the endothelial barrier function. The readings were also normalized to the untreated endothelial cells and therefore, only values with significant difference as compared to the untreated cells were plotted in the graphs to represent the real-time changes of the barrier function of the treated cells.***

**The following studies provide information on the sensitivity of ECIS system with regards to detecting low viral effects:**

**1. Mouse brain MEC infected with West Nile virus at MOI of 0.01 increased TEER up to 6 hours [1]**

**2. Reduced TEER was observed in Mouse Hepatitis Virus type 3-infected brain MEC at a MOI of 0.1 up to 72 hours [2]**

**3. Human Bocavirus-1 at the MOI of 0.001, 0.01, 0.1, 1, 10 and 100 reduced the TEER of human airway epithelial cells [3]**

**The studies above on the infection kinetics of different type of viruses on endothelial / epithelial cells provide information that the ECIS system is sensitive enough to detect small modulation of epithelial/endothelial cells infected with viruses at a relatively low MOIs.**

**Limitation of the ECIS has also been included in the last paragraph of the discussion.**

**References quoted:**

**[1] Lazear et al. (2015) Interferon-λ restricts West Nile virus neuroinvasion by tightening the blood-brain barrier, Sci Transl Med, 7(284): 284ra59.**

**[2] Bleau et al. (2015) Brain Invasion by Mouse Hepatitis Virus Depends on Impairment of Tight Junctions and Beta Interferon Production in Brain Microvascular Endothelial Cells, J Virol, 89(19): 9896-9908.**

**[3] Deng et al (2013) In Vitro Modeling of Human Bocavirus 1 Infection of Polarized Primary Human Airway Epithelia, J Virol, 87(7): 4097– 4102.**

Minor points:
Figures 7-10 could be easily grouped in one figure without losing clarity.

***Response: Figures 7-9 have been combined into Figure 7 and be divided into parts i), ii) and iii). To better discuss the results for pathway analysis as a separated outcome, Figure 10 was kept as a single figure and has been changed to Figure 8.***

Furthermore it would be important for the reader, to include in the volcano plots and in the Venn diagrams (i.e. Fig.8-9) full names of at least some of the top metabolites identified as significantly regulated by one or more classes. These figures as such are currently not conveying any meaningful information.

***Response: We agree with the reviewer’s comment, as such, the top regulated metabolites of each category have been added into the figures.***

Figures-7-13: The raw data and related statistical analysis of MS results should be presented in full in supplementary tables, to provide the reader with a mining-friendly resource. This is one of novelty points of the study, but it is virtually hidden as only selected candidates are presented in details in Fig.11-13.

***Responses: The raw data of metabolites present in serum samples from each category of DWOWS, DWWS and SD has been presented in supplementary S1 table (at end of manuscript).***
