# Supplementary material for: Correlation of host inflammatory cytokines and immune-related metabolites, but not viral NS1 protein, with disease severity of dengue virus infection
Source: PLoS One. 2020 Aug 7;15(8):e0237141. doi: 10.1371/journal.pone.0237141 (PMC7413495; doi:10.1371/journal.pone.0237141)
Supplement: Attachment — Submitted filename: Response to Reviewers 2.docx [file pone.0237141.s004.docx]

**Response to Reviewers:**

**Reviewer #2:** In manuscript titled “Correlation of viral NS1 protein, host inflammatory cytokines and immune-related metabolites with disease severity of dengue virus infection”, the authors tested sera from 40 dengue patients, and intended to evaluate the relation between the presence of NS1/host cytokines/host metabolites and the severity of dengue diseases. The overall conclusion is that no significant correlation has been found between the two. Therefore, their original intention to develop a potential marker for early diagnosis of dengue failed. The data collected in this manuscript can be valuable to understanding the dengue diseases and to the broader virology field if some of the essential controls are included in the experiments.

1) Introduction lacks a clear elaboration of the purpose of the study. It did not establish a clear rationale that supports the logic of testing NS1. For example, in lines 48-50, the authors said “Hence, this study aims to investigate the role of the viral non-structural 1 (NS1) protein, the inflammatory cytokines and the immune-related metabolites in contributing to the degree of severity of dengue virus infection”. However, the sentence before this conclusion is broad statement “The pathogenesis of severe dengue is theorized to be due to the intricate interactions between viral factors, host genetics and host immune activation” with no facts or details to explain why NS1 needs to be tested. More information should be included to elaborate the significance of why these experiments are done.

**Response: The role of NS1 in inducing vascular leakage have been described in the second paragraph. Nevertheless, the interactions between viral factors and host factors have been elaborated in the first paragraph to justify the rationale of investigating the correlation of viral factors and host factors with vascular leakage observed in patients with severe dengue.**

2) Some information introduced in the discussion, such as the first paragraph of discussion explaining ECIS, should be moved to introduction. It is strange to explain a method in discussion after the results have already been presented.

**Response: The paragraph discussing the principle of ECIS has been moved to introduction.**

3) Figure 1 showed purified NS1 increases vascular permeability in pulmonary MEC. Figures 2-4 did not show substantial differences when they used different groups of patient sera on different cells. To examine whether NS1 has the potential to become a diagnostic marker for dengue, an experiment that dilute the purified NS1 in uninfected human sera would give a clear answer. It is quite possible that certain sera composition blocks the NS1 effect.

**Response: The main objective of this manuscript is to assess the role of viral factor (NS1) and host factors (cytokines and metabolites) in contributing to the degree of vascular leakage in the microvascular endothelium during DENV infection, therefore, we focus on the sera of DENV infected patients with varying disease severity, which also contain NS1 protein at varying level. Since the findings showed that the serum NS1 level in dengue patients did not directly correlate to the extent of vascular leakage observed in MECs, we then proceeded to investigate the composition of the sera, in term of cytokines and metabolites, that might had overshadowed the effect of NS1. We have successfully identified some of the host proteins that were highly expressed in dengue patients and they might have played a bigger role as compared to NS1 in inducing vascular leakage. However, we would also like to highlight that no single factor alone should be concluded to be responsible for the progression of severe dengue, as the pathogenesis of dengue involves complex interactions between multiple factors. Nevertheless, the last paragraph of the introduction has been rephrased to better describe the main objectives of this study.**

4) The authors did metabolites analysis with the patient sera. However, without a control of healthy human sera, these data have no meaning for future analysis. Another possible control is to use human sera from another viral infection to show whether conclusion drawn from Figure 7-10 is dengue specific or not.

**Response: Control of healthy human sera was included for all the experiments of ECIS, cytokines and metabolomics profiling. The expression of cytokines and metabolites in dengue patients was compared against the expression profile of healthy individuals. The methodology of sample collection and selection, as well as the results of samples characteristics have been revised to better explain the samples used in this study.**

**Reviewer #3:** The manuscript Correlation of viral NS1 protein, host inflammatory cytokines and immune-related metabolites with disease severity of dengue virus infection presented by Sekaran SD et al, is an interesting study regarding an important cause of disease, dengue.
It is a laboratory study that analyze the effect of the addition of Dengue sera to Electrical cell-substrate impedance sensing (ECIS). Also, the cytokines and metabolic profile were measured.
I have several questions.

1. The primary and secondary dengue have differences in several geographic areas. The authors did not give any commentary about that.

**Response: The introduction has been revised to include the differences of primary and secondary infections in different geographic areas.**

2. The ECIS was performed with only 5 serum of dengue cases and none the original NS1 Ag was tested.

**Response: For ECIS, we investigated the effect of direct stimulation of NS1 antigen on microvascular endothelium, as well as the effect of NS1 in the presence of other host factors in the patient sera towards microvascular endothelial barrier function. A total of 20 samples were used, 5 from each of the categories of healthy individual, dengue without warning signs, dengue with warning signs and severe dengue to provide an insight to the pattern of the changes in the barrier function of four microvascular endothelial cell lines in response to NS1/sera stimulation.**

3. The cytokine profile were determined in only 40 serum (including from patients without alarm signs, with alarm signs or severe dengue). The authors need to include more patients. The same observations are done regarding the metabolic expression in dengue patients.

**Response: One of the limitations for this study was the small sample size of only 40 sera due to the difficulties in obtaining more samples from patients with severe dengue. Although the study has less statistical power, we were able to identify a few cytokines and metabolites that would provide insight on the role of host factors in the pathogenesis of dengue virus infection and possibly determine or predict disease severity that should be assessed with other approaches using a larger sample size in the future. This limitation has been discussed in the discussion.**

**Added revisions**

1) Please ensure that you refer to Figure 12 in your text as, if accepted, production will need this reference to link the reader to the figure.

**This figure is the graphical abstract that was requested when submitting. This has been referred as requested (Line: 573)**

2) Please include a legend for figure 12.

**This has been included.(Line 590)**

3) Please amend your current ethics statement to include the full name of the ethics committee/institutional review board(s) that approved your specific study**.**

**This has been amended and review board name given in full**

4) Please amend your current ethics statement to confirm that your named institutional review board or ethics committee specifically approved this study.

**This has been amended and review board name given in full**
