# Supplementary material for: Correlation of host inflammatory cytokines and immune-related metabolites, but not viral NS1 protein, with disease severity of dengue virus infection
Source: PLoS One. 2020 Aug 7;15(8):e0237141. doi: 10.1371/journal.pone.0237141 (PMC7413495; doi:10.1371/journal.pone.0237141)
Supplement: Attachment — Submitted filename: Response to Reviewers.docx [file pone.0237141.s005.docx]

**Response to Reviewers**

**Reviewer #2:**

1) The authors addressed my questions. However, the newly added Figure 12 cannot be found in the paper.

**Response: Figure 12 was named after the graphical abstract; it is now being renamed as Graphical abstract and was uploaded separately.**

2) Now that it is clearly described in the materials and methods that the 40 samples include 30 from patients and 10 from healthy people, the sample size is really small.

**Response: This limitation is now highlighted in the abstract to state that the findings deserved to be confirmed or extended using independent cohort with a bigger sample size.**

**Reviewer #3:**

The manuscript Correlation of viral NS1 protein, host inflammatory cytokines and immune-related metabolites with disease severity of dengue virus infection presented by Sekaran SD et al, is an interesting study regarding an important cause of disease, dengue.
It is a laboratory study that analyzes the effect of the addition of Dengue sera to Electrical cell-substrate impedance sensing (ECIS). Also, the cytokines and metabolic profile were measured.
I have several questions.

1) Although the authors say that they have include the differences of primary and secondary infections in different geographic areas, in the results the differences are scarce.

**Response: The differences of primary and secondary infections in different geographic areas were included in the introduction as supplementary information to the topic. However, we did not include the differences between primary and secondary infections in the experiment planning as we would like to focus on the categories of disease severity.**

2) Moreover, the cytokines were determined in only 40 serum samples from four categories, HC, DWOWS, DWWS and SD. This explained that only eight serum were analyzed by categories. The metabolic expressions of dengue patients have the same difficulty. Only 40 sera were studied, corresponding to four categories.

**Response: The limitation of small sample size has been included in the discussion, as we feel that the exploratory findings merits further investigation, this limitation is now highlighted in the abstract to state that the findings deserved to be confirmed or extended using independent cohort with a bigger sample size.**
